# Non-Venereal Sclerosing Lymphangitis of the Penis in a 35-Year-Old Saudi Male: A Case Report

**DOI:** 10.7759/cureus.31087

**Published:** 2022-11-04

**Authors:** Mishari T Alrubaiaan, Moteb Alotaibi, Khalid A Alekrish

**Affiliations:** 1 College of Medicine, King Saud Bin Abdulaziz University for Health Sciences, Riyadh, SAU; 2 Department of Dermatology, Unaizah College of Medicine and Medical Sciences, Qassim University, Unaizah, SAU; 3 College of Medicine, King Saud University, Riyadh, SAU

**Keywords:** penis, std, venereology, dermatology, sclerosing lymphangitis

## Abstract

Sclerosing lymphangitis is a rare penile lesion characterized by a cord-like, firm swelling at the penile coronal sulcus. It affects males between the ages of 30 and 40 and usually resolves spontaneously. Due to the rarity of this condition, we decided to report this case. Herein, we present a case of a 35-year-old male that was evaluated for a painless, cord-like, penile lesion that enlarges during erection, characteristic of sclerosing lymphangitis.

## Introduction

Sclerosing lymphangitis is a benign, under-reported illness of the penis that manifests in the form of an asymptomatic firm cord-like swelling around the coronal sulcus of the penis, often affecting males in their third and fourth decades of life. Hoffman described sclerosing lymphangitis of the penis for the first time in 1923 [1]. The condition was defined as a firm, worm-like subcutaneous lesion surrounding the penile shaft known as “gonorrheal pseudo chancre” [1]. The ailment was later described by the author as “non-venereal plastic lymphangitis of the coronary sulcus of the penis with confined edema,” indicating that it is non-venereal in origin [2]. Since then, other cases-albeit with different terminology-have been documented. Sclerosing lymphangitis of the penis, which will be described herein, is one of these terminologies that has been used the most often [3].

Sclerosing lymphangitis is attributed to the blockage of lymphatic vessels, leading to a painless, firm, translucent, linear, cord-like swelling around the coronal sulcus of the penis [3]. It manifests commonly during the third and fourth decades of life, with a reported range from 18 to 66 years of age [4-6]. Although the exact causes are still unknown, repeated trauma through vigorous sexual activity is thought to be one of the most common risk factors [7]. Moreover, circumcision and sexually transmitted infections may also be causal factors [8]. Because of its asymptomatic and self-limiting nature, sclerosing lymphangitis is considered an under-reported medical condition [9].

Herein, we describe the medical history and physical examination findings of a thirty-five-year-old male where the diagnosis of sclerosing lymphangitis was established. The rarity of the described condition was the primary motivator for this case report.

## Case presentation

A 35-year-old, sexually active, circumcised man was evaluated at the dermatology and venereology clinic for a painless penile lesion that had appeared two days prior. He reported having sexual intercourse one to two times per week; nevertheless, his last sexual activity was one month before lesion onset, during which he used sildenafil citrate at a dose of 50 mg. Furthermore, he denied any history of penile manipulation or trauma, vigorous sexual behavior, penile or scrotal swellings, urethral discharge, genital ulceration, or previous similar lesions. His past medical and family history were unremarkable, and he was otherwise healthy.

**Figure 1:**
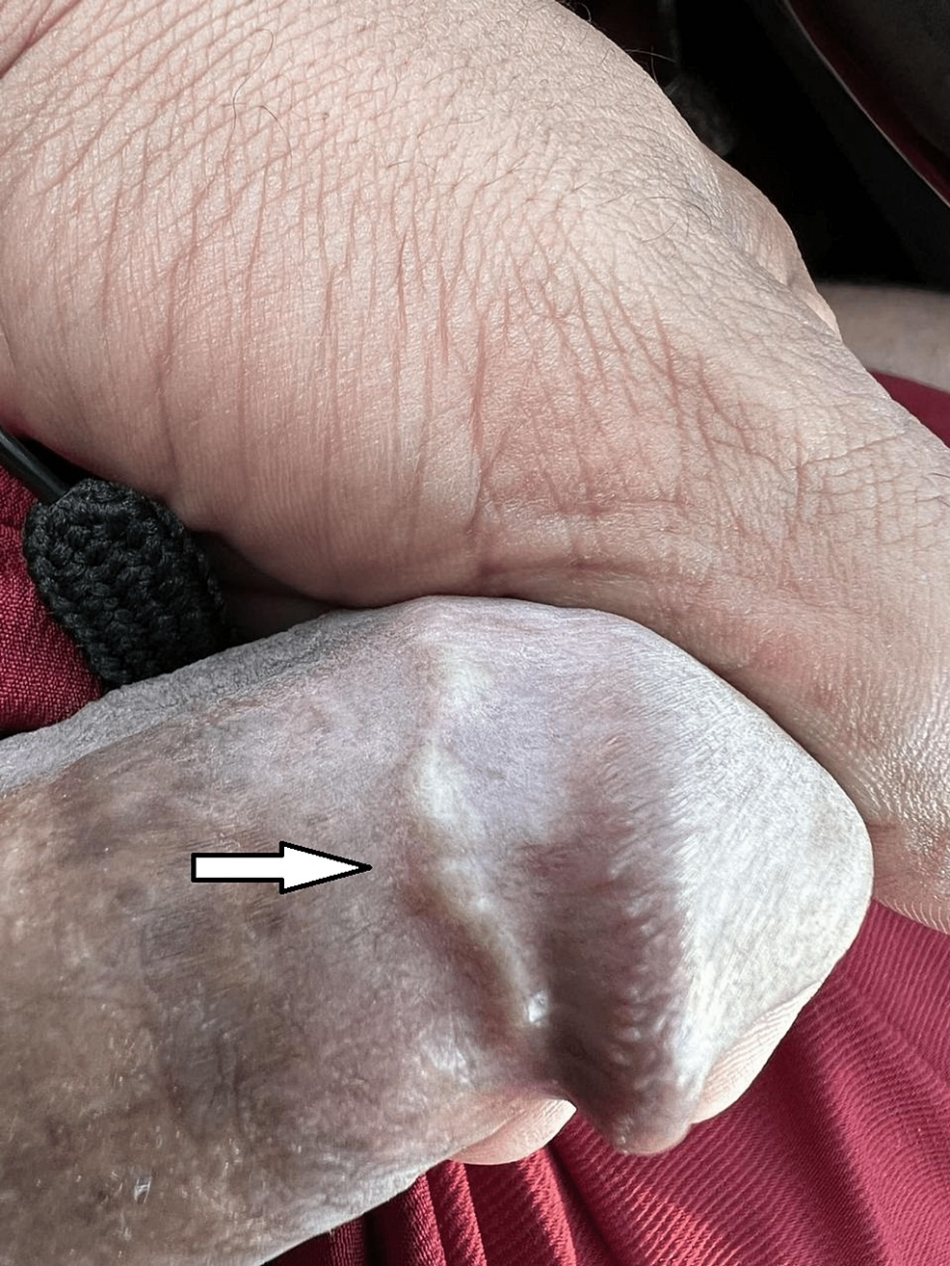
Linear induration near the coronal sulcus, which becomes more prominent upon erection

On physical examination, the penile lesion appeared as a single, three-millimeter, skin-colored, cord-like induration on the lateral aspect of the penis, partially encircling the coronal sulcus (Figure 1). Upon palpation, the lesion was firm and non-tender. The skin overlying the cord was freely mobile, and no signs of inflammation were noted. Moreover, the dorsum of the penis was free of any similar lesion.

Screening tests for Sexually Transmitted Infections (STI), including syphilis, chlamydia, gonorrhea, human immunodeficiency virus, herpes simplex viruses, and hepatitis B and C viruses, were all unremarkable.

Based on the unique clinical presentation and after considering other potential differential diagnoses, the diagnosis of non-venereal sclerosing lymphangitis was established. Sexual abstinence alongside oral prednisolone at a dose of 15 mg daily for seven days successfully cleared the lesion.

## Discussion

Sclerosing lymphangitis, also known as “Non-Venereal Plastic Lymphangitis of the Penis,” is a rare, benign condition first described by Hoffman in 1923 [1], where an asymptomatic swelling arises around the coronal sulcus of the penis [1,9-11]. The diagnosis of sclerosing lymphangitis is often made on clinical grounds where swelling can be visualized on the penile shaft in a circumcised patient without systemic symptoms [9].

It was found that men who are sexually active in their third or fourth decades of life are often affected. On the other hand, several earlier instances in their sixth decade have been documented [4-6]. It is still unknown what the specific underlying cause is. Yet, most articles in the literature placed particular emphasis on injuries experienced during prolonged, frequent, and intense sexual activity or penile manipulation that results in venous or lymphatic blockage [7,8]. Although it is generally agreed upon that sclerosing lymphangitis is not brought on by a particular infectious organism, several sexually transmitted illnesses have been linked to this condition in prior publications, specifically, Chlamydia, Herpes, and Syphilis [12-14]. However, in this case, STI screening for the patient was performed, and the result was unremarkable. Furthermore, Broadus et al. suggested that in circumcised men, circumferential scaring may be a contributing factor, which is the case with our patients [15]. Additionally, a recent report suggested that increased plasma concentration of tadalafil could be a precipitating factor in the development of sclerosing lymphangitis in the existence of anatomical variation in the venous arcade [16]. It is unknown whether sildenafil carries the same risk as tadalafil in precipitating sclerosing lymphangitis.

Clinically speaking, sclerosing lymphangitis is characterized by the abrupt emergence of small, often asymptomatic, firm, non-tender, cord-like swelling around the coronal sulcus of the penis [4]. It may, however, sometimes show up farther down the penile shaft. Typically, the skin above is readily movable and free of any accompanying erosions or ulcerations [9]. Even though these lesions have no symptoms, some individuals may feel slight pain or discomfort, particularly during erection [4-8]. Early diagnosis relies only on a thorough clinical examination and accurate medical history gathering; a biopsy or ultrasonography has no significance and has no bearing on the course of therapy [9]. Moreover, Sclerosing lymphangitis is a self-limiting disease that tends to regress spontaneously over two months. The resolution, however, can be accelerated by sexual abstinence [17]. This patient showed a rapid improvement within a week of being on prednisolone 15 mg daily.

Sclerosing lymphangitis is often very difficult to distinguish from a similar condition, Penile Mondor’s Disease (PMD), a superficial thrombophlebitis of the dorsal vein. Clinically, a painful, rigid cord-like structure appears on the dorsum of the penis. In contrast to sclerosing lymphangitis, which is freely movable, the cord is often attached to the overlaying skin in the case of PMD [18]. The diagnosis of PMD can also be made based on clinical findings. However, a doppler ultrasound can confirm the diagnosis by revealing echogenic, non-compressible veins while being normal in sclerosing lymphangitis. Moreover, on biopsy, an obstruction of the lumen of the vein can indicate PMD, whereas hypertrophy and sclerosis of lymphatic vessels are characteristically observed in sclerosing lymphangitis [19]. Sexual abstinence, together with or without the use of non-steroidal anti-inflammatory drugs, is often recommended as a treatment. Vein stripping may be recommended as a medical intervention to treat PMD in situations of advanced illness when no improvement has been shown after 10 weeks [9].

When assessing a patient for an asymptomatic penile lesion, we recommended that clinicians consider sclerosing lymphangitis as a possibility, particularly if the lesion is augmented by erection, as this condition can be easily treated with no resulting complications. Furthermore, we encourage physicians to obtain a full STI panel where sclerosing lymphangitis is diagnosed.

## Conclusions

Sclerosing lymphangitis is a condition of undetermined cause and is treated conservatively. To the best of our knowledge, this is the first case of sclerosing lymphangitis to be reported in the region. We encourage dermatologists and venereologists to beware of sclerosing lymphangitis as a possible diagnosis in any middle-aged male who presents with a painless, linear, penile lesion, as such diagnosis can be indicative of underlying sexually transmitted diseases.
